# Thymoquinone Protects against Myocardial Ischemic Injury by Mitigating Oxidative Stress and Inflammation

**DOI:** 10.1155/2015/143629

**Published:** 2015-05-25

**Authors:** Shreesh Ojha, Sheikh Azimullah, Rajesh Mohanraj, Charu Sharma, Javed Yasin, Dharamvir S. Arya, Abdu Adem

**Affiliations:** ^1^Department of Pharmacology and Therapeutics, College of Medicine and Health Sciences, United Arab Emirates University, P.O. Box 17666, Al Ain, Abu Dhabi, UAE; ^2^Department of Internal Medicine, College of Medicine and Health Sciences, United Arab Emirates University, P.O. Box 17666, Al Ain, Abu Dhabi, UAE; ^3^Department of Pharmacology, All India Institute of Medical Sciences, New Delhi 29, India

## Abstract

The present study was aimed at investigating the cardioprotective activity of thymoquinone (TMQ), an active principle of the herb,* Nigella sativa*, which is used for the management of various diseases. The present study examined the cardioprotective effect of TMQ in isoproterenol- (ISP-) induced myocardial infarction in rats. Myocardial infarction was induced by two subcutaneous injections of ISP (85 mg/kg) at an interval of 24 hr. TMQ (20 mg/kg) was administered orally for 21 days. ISP-treated rats showed depletion of antioxidants and marker enzymes from myocardium along with lipid peroxidation and enhanced levels of proinflammatory cytokines. ISP also induced histopathological alterations in myocardium. Treatment with TMQ prevented the depletion of endogenous antioxidants and myocyte injury marker enzymes and inhibited lipid peroxidation as well as reducing the levels of proinflammatory cytokines. TMQ pretreatment also reduced myonecrosis, edema, and infiltration of inflammatory cells and showed preservation of cardiomyocytes histoarchitecture. The present study results demonstrate that TMQ exerts cardioprotective effect by mitigating oxidative stress, augmenting endogenous antioxidants, and maintaining structural integrity. The results of the present study indicate that TMQ may serve as an excellent agent alone or as adjuvant to prevent the onset and progression of myocardial injury.

## 1. Introduction

Myocardial infarction (MI) is a major form of ischemic heart disease, characterized by an imbalance of coronary blood supply and myocardial demand which results in ischemia and myocardial death. Experimental and clinical studies have shown that, during ischemic injury, produced oxidative stress plays a key role in the development of MI [1, 2]. In ischemic tissues, the oxygen-free radicals have been implicated in oxidative chain reactions, which damage the cell membrane and subcellular structures containing phospholipids and proteins. These reactions further cause phospholipid peroxidation and subsequently lead to functional, structural, and metabolic alterations in the heart [2].

A large number of epidemiological, clinical, and experimental studies have demonstrated that the use of antioxidants as a preventive approach may limit the infarct size and attenuate myocardial dysfunction as well as slowing down the progression and consequences of MI [3–6]. Antioxidants not only suppress the formation of reactive oxygen species (ROS) and free radical generation and or augmentation of endogenous antioxidant enzymes but also modulate heart function [2–4]. The central role of ROS in the pathophysiology of MI has been confirmed by the ability of antioxidants to reduce ischemic injury in the animal model of isoproterenol- (ISP-) induced MI [7–9]. The pathophysiological and morphological changes in myocardium of ISP administered to rats closely resemble human MI [7, 8]. ISP, a synthetic catecholamine and *β*-adrenergic agonist, is well known to produce MI in rats as a result of disturbance in physiological balance between production of highly cytotoxic free radicals and antioxidative defense network [9, 10]. Compromised antioxidant defense leads to metabolic and functional impairment and membrane permeability changes consequent to lipid peroxidation and ultimately irreversible damage to the myocyte membrane [9–11].

Thymoquinone, an aromatic ketone, is a major constituent in the seeds of* Nigella sativa*, known as black cumin in English and* “Habba Al-Sauda”* and* “Habba Al-Barakah”* in Arabic [12, 13]. The seeds of black cumin are frequently used in Middle Eastern countries in traditional medicine for the treatment of many ailments as well as for the improvement in general health and well-being [12]. The cardiovascular benefits of black cumin were recently reviewed and it was concluded that black seed has a wider therapeutic potential especially in cardiovascular diseases [12]. The treatment with black cumin extract has been reported to decrease cyclosporine A [14], cyclophosphamide [15], and doxorubicin [16] induced myocardial injury in animal studies. In recent years, the active component TMQ is regarded as a potent antioxidant [17] and has anti-inflammatory [17] properties and showed anticancer [17], nephroprotective [18], hepatoprotective [19], and neuroprotective [20] activities. TMQ has been reported to cause a dose-dependent decrease in the arterial blood pressure and heart rate in spontaneously hypertensive rats [21] and doxorubicin-induced cardiotoxicity [15]. Additionally, TMQ has been reported to show cardiovascular relaxant activity by modulating atrial force and rate of contraction mediated by blockade of voltage gated Ca^+2^ channels in* in vitro* studies [13].

In the present study, we investigated the cardioprotective effect of TMQ against isoproterenol-induced myocardial injury, a clinically relevant animal model measuring the markers of oxidative stress, inflammation, and myocyte injury. Furthermore, to support our findings, we also examined the effects of TMQ on histopathological changes in the myocardium.

## 2. Material and Methods

### 2.1. Experimental Animals

Adult male Wistar rats (230–250 g) were obtained from the animal research facility of College of Medicine and Health Sciences, United Arab Emirates University, Al Ain, UAE. The experimental protocols were approved by the Animal Ethics Committee of College of Medicine and Health Sciences, United Arab Emirates University, Al Ain, UAE. The animals were housed under standard laboratory conditions. The animals had free access to commercially available standard rodent diet and water and were fed* ad libitum*. A maximum of four rats were housed per cage and acclimatized to the laboratory conditions prior to the commencement of the experiment.

### 2.2. Drugs and Chemicals

**Figure 1:**
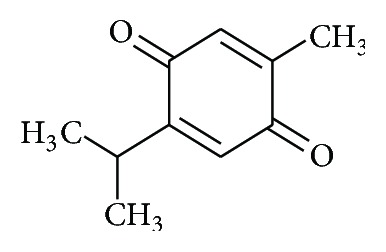
Chemical structure of thymoquinone.

Isoproterenol (1-(3,4-dihydroxyphenyl)-2-isopropylaminoethanol hydrochloride, molecular weight: 247.72) and TMQ (2-isopropyl-5-methyl-1, 4-benzoquinone; TMQ) (CAS number 490-91-5, molecular formula C_10_H_12_O_2_, molecular weight 164.20, and purity 99%, Figure 1) were procured from Sigma Aldrich (St. Louis, MO, USA). The chemicals and reagents including bovine serum albumin (BSA), 5-sulfosalicylic acid (SSA), naphthylene diamine dihydrochloride, sulphanilamide, phosphoric acid, HEPES, sucrose, 1,4-dithiothreitol (DTT), CHAPS, sodium chloride, protease inhibitors, phenyl methyl sulfonyl fluoride (PMSF), Tween-20, sodium nitrate, 3,3,5,5′-tetramethyl benzidine (TMB), and reduced form of glutathione (GSH) assay kit were purchased from Sigma Aldrich. The enzyme linked immunosorbent assay (ELISA) kit was obtained from R&D Systems, USA. The aspartate transaminase (AST), alanine transaminase (ALT), creatine kinase (CK), and lactate dehydrogenase (LDH) kits were procured form Roche Diagnostics, USA.

### 2.3. Induction of Experimental MI in Rats

Experimental MI in rats was induced by administering ISP (85 mg/kg body weight) as reported in earlier literature including our study [10]. ISP was dissolved in physiological saline and injected subcutaneously for two consecutive days at the interval of 24 hrs. The animals injected with ISP provide a relatively inexpensive and easily accessible rodent model that mimics the natural history and metabolic characteristics of patients with MI [7, 8]. ISP, a synthetic nonselective *β*-adrenoceptor agonist, is well-accepted noninvasive rat model to induce MI and is widely used to evaluate cardioprotective effects of pharmacological agents against MI [9, 10]. The pathophysiological and morphological changes of ISP-induced myocardial necrosis are similar to those observed in humans with MI [8].

### 2.4. Experimental Groups and Design

The animals were randomly divided into four experimental groups, each containing eight rats. Group I (normal group; control) animals received normal saline using intragastric tube for 21 days and on the 20th and 21st day saline was administered (500 *μ*L/rat, s.c.) at an interval of 24 hr. Group II (ISP control; ISP) animals received normal saline using intragastric tube for 21 days and on the 20th and 21st day ISP was administered (85 mg/kg, s.c.) at 24 hr interval. Group III (TMQ control; TMQ) animals received only TMQ (20 mg/kg/day) orally using intragastric tube for 21 days and on the 20th and 21st day saline was administered (500 *μ*L/rat, s.c.) at an interval of 24 hr. Group VI (TMQ + ISP) animals received TMQ (20 mg/kg/day) orally using intragastric tube for 21 days along with concurrent administration of ISP (85 mg/kg, s.c. at 24 hr interval) on days 20 and 21. The dose of the TMQ (20 mg/kg) and the duration of pretreatment (21 days) of TMQ were based on a pilot study in our laboratory (data not shown). During the experimental period, the body weights of the rats were monitored at regular intervals.

At the end of the experimental period, that is, 24 hrs after the second injection of ISP or 48 hrs after the first injection of ISP, the rats were euthanized under the influence of the anesthetic; sodium pentobarbitone and the blood was collected for the determination of myocyte injury marker enzymes such as AST, ALT, CK, and LDH. The heart was excised and processed for the estimation of biochemical markers of oxidative stress, superoxide dismutase (SOD), catalase, nitric oxide (NO) and reduced glutathione (GSH), malondialdehyde (MDA), and proinflammatory cytokines, interleukin-1*β* (IL-1*β*), interleukin-6 (IL-6), and tumor necrosis factor-alfa (TNF-*α*) using kits.

### 2.5. Preparation of Heart Tissue Homogenate

The heart was excised, washed in ice-cold phosphate buffer saline, and minced into fine fragments followed by homogenization using a polytron homogenizer (IKA Laboratory, Germany), with 5 volumes of ice-cold buffer containing 100 mM HEPES, pH 7.5, 10% sucrose, 10 mM DTT, 0.1% CHAPS, 150 mM NaCl, protease inhibitors tablet, and 1 mM PMSF. The aliquots were used for the estimation of GSH and MDA. Further, the samples were centrifuged at 10000 g for 10 min and the obtained supernatant was removed and stored at −80°C for the estimation of cytokines using ELISA kits.

### 2.6. Determination of Oxidative Stress Markers

The levels of GSH, MDA, and NO were determined using commercially available kits in heart homogenates.

#### 2.6.1. Estimation of Reduced Glutathione (GSH)

The GSH content in heart homogenate was estimated following manufacturer protocol of the assay kit (Sigma Aldrich, MO, USA). Briefly, the measurement of GSH uses a kinetic assay in which catalytic amounts (nmoles) of GSH cause a continuous reduction of 5,5-dithiobis(2-nitrobenzoic acid) to nitrobenzoic acid (TNB) and the glutathione disulfide (GSSG) formed was recycled by glutathione reductase and NADPH. The yellow color product, 5-thio-2-TNB, was measured spectrophotometrically at 412 within 5 min of 5,5-dithiobis(2-nitrobenzoic acid) addition, against a blank with no homogenate. GSH concentration was expressed as *μ*M of GSH/mg of tissue.

#### 2.6.2. Estimation of Malondialdehyde (MDA)

The lipid peroxidation product, MDA in the heart homogenate from each group was measured using the MDA assay kit purchased from Northwest Life Science Specialties (WA, USA). Briefly, the assay is based on the reaction of MDA with thiobarbituric acid (TBA) forming a MDA-TBA adduct that absorbs strongly at 532 nm. Briefly, the deproteinated tissue sample was added to 1 M phosphoric acid and butylated hydroxyltoluene in ethanol and then the mixture was heated at 60°C for 60 min. The suspension was cooled to room temperature, centrifuged at 10000 g for 2-3 min, and the pink colored supernatant was taken for spectroscopic measurements at 532 nm for the assay of MDA. The concentration of MDA was expressed as *μ*M/mg of tissue.

#### 2.6.3. Estimation of Nitric Oxide (NO)

Accumulation of nitric oxide in myocardial tissues was determined according to the Griess reagent method (0.2% naphthylene diamine dihydrochloride and 2% sulphanilamide in 5% phosphoric acid). Briefly, 100 *μ*L of sample was mixed with an equal volume of Griess reagent and incubated at room temperature for 10–15 min. The absorbance at 492 nm was measured in an automated microplate reader (Tecan Group Limited, Männedorf, Switzerland). The nitrite concentration was quantitated using NaNO_2_ as standard and was expressed as *μ*M/mg of tissue.

### 2.7. Determination of Proinflammatory Cytokines in Heart

Enzyme immunoassay of IL-1*β*, IL-6, and TNF-*α* in heart homogenate was performed by using sandwich R&D duo set ELISA kit (Minneapolis, USA). Briefly, the wells of a 96-well microtiter plate were coated with respective primary antibody in phosphate buffer saline (PBS) (100 *μ*L/well) overnight at room temperature, washed with phosphate-buffered saline containing 0.05% Tween-20 (PBST), and then blocked with 1% bovine serum albumin in PBS for one hr. After washing, plates were incubated with serum, heart homogenates, and respective standards for 2 hrs. After washing with PBST, a detection antibody was added for 2 hrs and 100 *μ*L of HRP was added for half an hr, after the washing. The TMB-ELISA substrate was added and the color intensity read at 450 nm with a microplate reader (Tecan Ltd., Männedorf, Switzerland). The cytokines levels were expressed as pg/mg of tissue.

### 2.8. Determination of Myocardial Infarct Size

Myocardial necrosis is also detected by direct staining method using TTC (triphenyl tetrazolium chloride) dye, which forms a red formazan precipitate with LDH of the viable myocardial tissue, whereas the infarcted myocardium fails to stain with TTC [22]. The 2 mm tissue slices were cut from the heart and incubated at 37°C for 30 min in 1% TTC stain before keeping them in 10% neutralized buffered formalin for overnight. The infarct areas were analyzed using Image J software (NIH, USA).

### 2.9. Histopathological Studies

Tissues fixed in buffer formalin were embedded in paraffin and serial sections (5 *μ*m thick) were cut using microtome (Leica RM 2125, Germany). Each section was stained with hematoxylin and eosin (H&E). The sections were examined under the light microscope (Olympus, Germany) and digital images were acquired. The degree of necrosis was graded as follows: −, absence of inflammation, edema, and necrosis; +, focal areas of inflammation, edema, and necrosis; ++, patchy areas of inflammation, edema, and necrosis; +++, confluent areas of inflammation, edema, and necrosis; ++++, massive areas of inflammation, edema, and necrosis.

### 2.10. Statistical Analysis

Data was analyzed statistically using SPSS 19.0 software. The means of the data are presented with the standard error of mean (SEM). The results were analyzed using one-way ANOVA to determine the significance of the mean between the groups. The values of *P* < 0.05 were considered significant.

## 3. Results

### 3.1. Effect of Only Thymoquinone Only (*Per Se*) Treatment

TMQ* per se* treatment (20 mg/kg) did not show any significant change in biochemical and histological parameters as compared to vehicle control group. Though, a significant decrease in the SOD activity was observed in only TMQ group compared to the vehicle group.

### 3.2. Effect of Thymoquinone on Heart Weight to Body Weight Ratio

**Figure 2:**
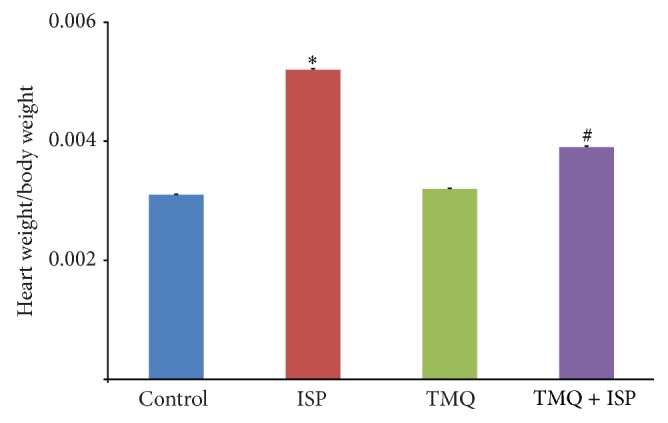
Effect of thymoquinone on heart weight/body weight ratio. Results are mean ± SEM (*n* = 6); ^*^ *P* < 0.05 versus ISP and ^#^ *P* < 0.05 versus TMQ + ISP.

ISP challenge produced a significant increase in heart weight/body weight ratio in comparison with control rats (Figure 2). However, TMQ treatment caused a significant decrease in the heart weight/body weight ratio when compared to ISP control group.

### 3.3. Effect of Thymoquinone on Antioxidant Enzymes

**Figure 3:**
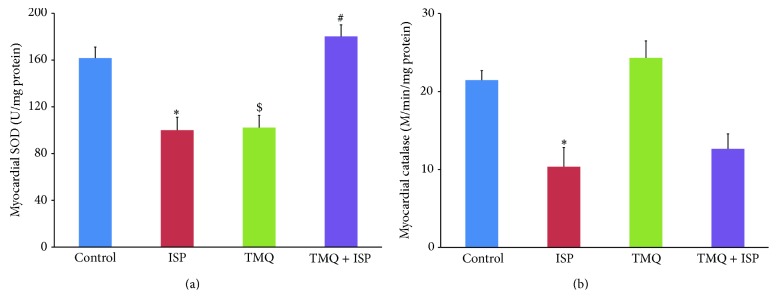
Effect of thymoquinone on myocardial levels of (a) SOD and (b) catalase. Results are mean ± SEM (*n* = 6); ^*^ *P* < 0.05 versus ISP, ^$^ *P* < 0.05 versus normal, and ^#^ *P* < 0.05 versus TMQ + ISP.

The changes in the antioxidant enzymes, SOD and catalase in the rats of different experimental groups, are represented in Figures 3(a) and 3(b). A significant decrease in the activities of SOD and catalase was observed in ISP administered rats as compared to control group. Following treatment with TMQ, a significant improvement in myocardial SOD activity was observed in comparison with ISP control group. However, TMQ treatment fails to improve the catalase activity significantly in comparison with ISP control group.

### 3.4. Effect of Thymoquinone on Glutathione

**Figure 4:**
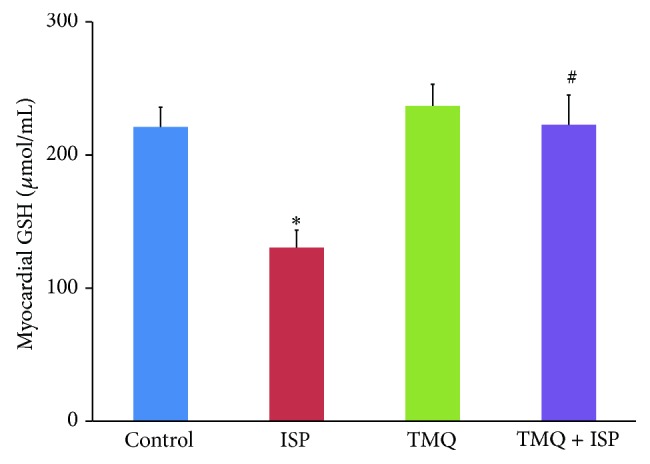
Effect of thymoquinone on myocardial levels of GSH. Results are mean ± SEM (*n* = 6); ^*^ *P* < 0.05 versus ISP and ^#^ *P* < 0.05 versus TMQ + ISP.

The rats administered ISP showed a significant decrease in the myocardial GSH level when compared to the control group (Figure 4), whereas TMQ treatment increased the level of GSH in comparison with ISP control group (Figure 4).

### 3.5. Effect of Thymoquinone on Lipid Peroxidation

**Figure 5:**
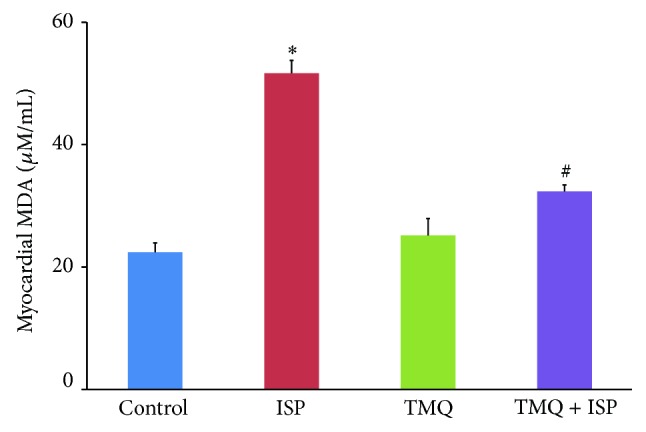
Effect of thymoquinone on myocardial levels of MDA. Results are mean ± SEM (*n* = 6); ^*^ *P* < 0.05 versus ISP and ^#^ *P* < 0.05 versus TMQ + ISP.

The rats administered ISP showed a significant increase in the lipid peroxidation product and MDA in heart when compared to control group (Figure 5). However, treatment with TMQ has significantly inhibited the level of MDA as compared to ISP control group (Figure 5).

### 3.6. Effect of Thymoquinone on Nitric Oxide

**Figure 6:**
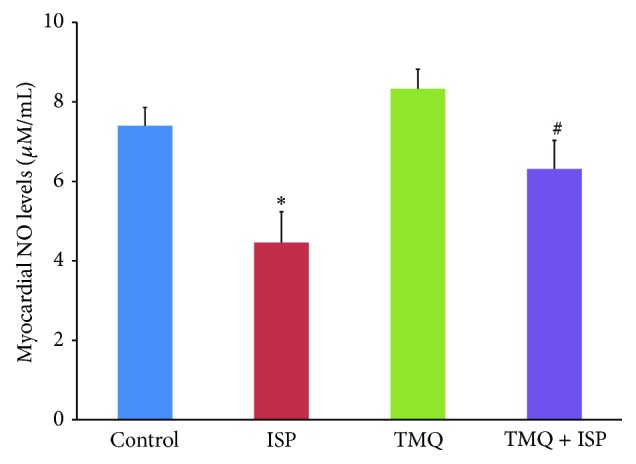
Effect of thymoquinone on myocardial levels of NO. Results are means ± SEM (*n* = 6); ^*^ *P* < 0.05 versus ISP and ^#^ *P* < 0.05 versus TMQ + ISP.

The myocardial content of NO was significantly decreased in ISP-challenged rats compared to control group (Figure 6). However, treatment with TMQ has significantly increased the NO levels in heart tissue as compared to the ISP control group (Figure 6).

### 3.7. Effect of Thymoquinone on Myocyte Injury Markers

**Figure 7:**
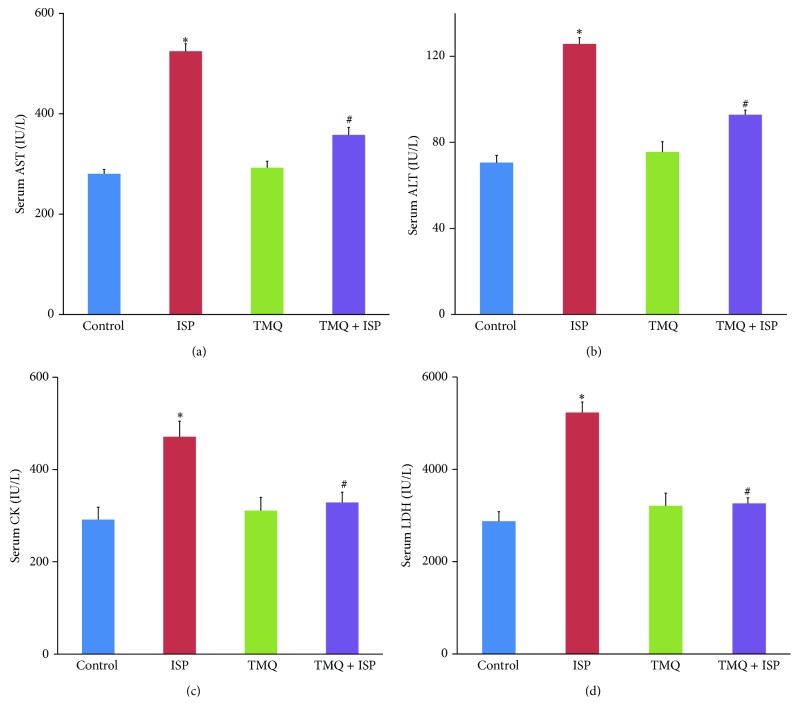
Effect of thymoquinone on serum levels of (a) AST, (b) ALT, (c) CK, and (d) LDH. Results are mean ± SEM (*n* = 6); ^*^ *P* < 0.05 versus ISP and ^#^ *P* < 0.05 versus TMQ + ISP.

As shown in Figures 7(a)–7(d), the levels of diagnostic markers of myocardial injury markers, AST, ALT, CK, and LDH, were significantly increased in the serum of ISP administered rats in comparison with control group. However, rats pretreated with TMQ showed significant reduction of the levels of AST, ALT, LDH, and CK when compared to the ISP control group.

### 3.8. Effect of Thymoquinone on Proinflammatory Cytokines

**Figure 8:**
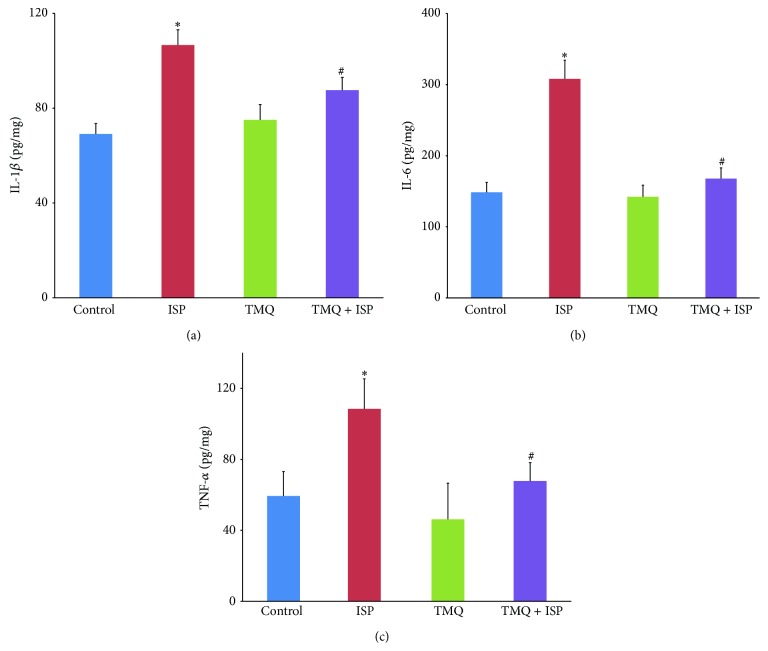
Effect of thymoquinone on myocardial levels of (a) IL-1*β*, (b) IL-6, and (c) TNF-*α*. Results are mean ± SEM (*n* = 6); ^*^ *P* < 0.05 versus ISP and ^#^ *P* < 0.05 versus TMQ + ISP.

Figures 8(a)–8(c) represent the levels of the proinflammatory cytokines, IL-1*β*, IL-6, and TNF-*α*, in the heart of different experimental groups. There was a significant increase in the level of IL-1*β*, IL-6, and TNF-*α* in the heart of ISP-challenged rats when compared to normal control group. However, on treatment with TMQ, a significant decline in the myocardial levels of IL-1*β*, IL-6, and TNF-*α* was observed when compared to the ISP control group.

### 3.9. Effect of Thymoquinone on Infarct Area

**Figure 9:**
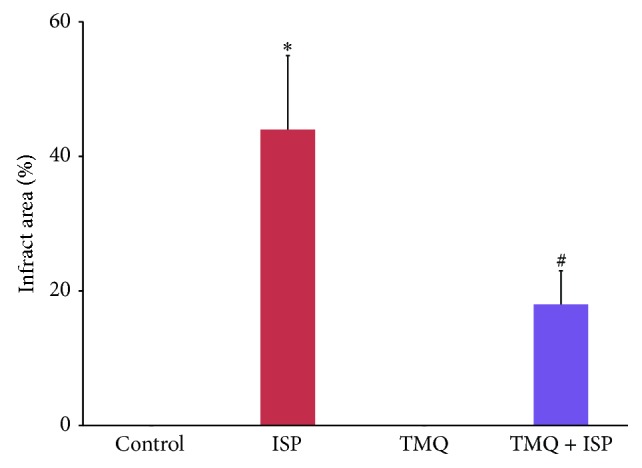
Effect of thymoquinone on myocardial infarct size in TTC staining. ^*^ *P* < 0.05 versus ISP and ^#^ *P* < 0.05 versus TMQ + ISP.

The effect of TMQ on macroscopic enzyme assay as analyzed by TTC method indicates marked changes in the area of infarction (Figure 9). While in ISP-induced rats large unstained region with more necrotic patches were observed, the heart slice of the ISP-challenged animal which received TMQ exhibited tissue viability with less necrotic tissues.

### 3.10. Effect of Thymoquinone on Histopathology

**Figure 10:**
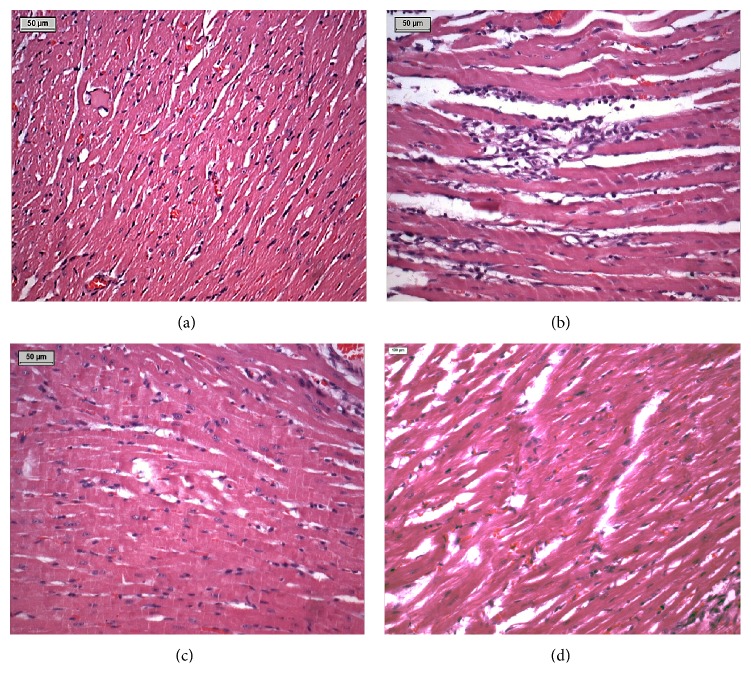
Light microscopic changes of myocardium showing (a) normal architecture of myocardium in vehicle control group, (b) focal necrosis of myofibrils and edema with infiltration of inflammatory cells and extravasations of red blood cells in ISP control group, (c) normal histoarchitecture of only TMQ treated group, and (d) lessened myocardial necrosis, edema, and infiltration of inflammatory cells in TMQ + ISP-treated group.

**Figure 11:**
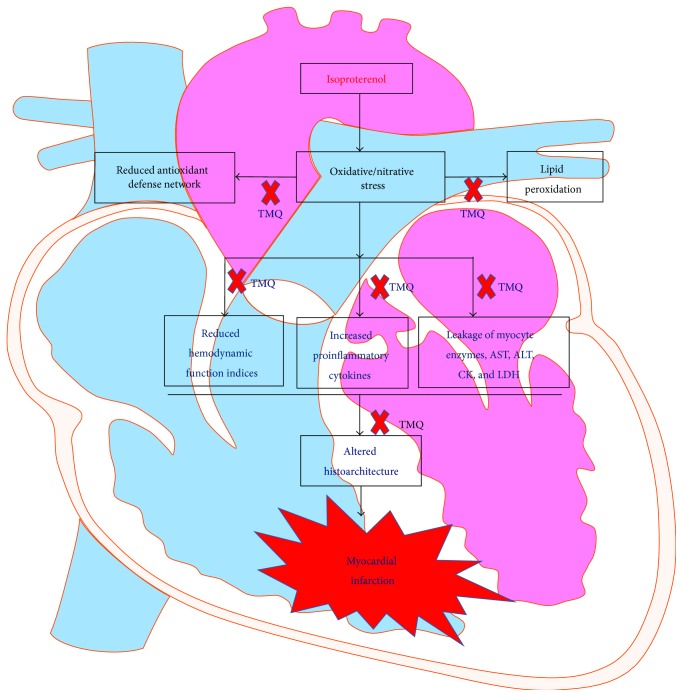
Schematic presentation of cardioprotective efficacy of thymoquinone in isoproterenol-induced myocardial infarction.

Figures 10(a)–10(d) show the light micrograph of the myocardium of different experimental groups. The light microscopic observations of myocardial histoarchitecture were qualitatively graded on the basis of myonecrosis, inflammatory cells, and edema (Table 1). The myocardium of vehicle control group showed a normal histoarchitecture (Figure 10(a)). Myocardium of ISP control rats showed marked necrosis of myofibers with cell infiltration, edema, and increased connective tissue among myocardial fibers along with extravasations of red blood cells (Figure 10(b)). Rats which received TMQ (20 mg/kg)* per se* did not show any adverse effect on myocardial histology (Figure 10(c)). On the other hand, treatment with TMQ in ISP-challenged rats showed very mild degree of myonecrosis, edema, and inflammation with a close resemblance to the normal myocardial histoarchitecture of vehicle control group (Figure 10(d)).  Figure 11 depicts a schema of the possible cardioprotective mechanism of TMQ.

## 4. Discussion

The results of present study demonstrate the protective effect of TMQ in ISP-induced MI in rats. Treatment with TMQ in ISP-challenged rats showed significant improvement in heart weight/body weight ratio and reduced myocardial infarct area and serum levels of myocyte marker enzymes along with restoration of antioxidants with concomitant reduction in lipid peroxidation. Along with consistent improvement in biochemical parameters, TMQ significantly preserved the myocardial histoarchitecture.

The animal model of ISP-induced myocardial injury recapitulates major occurring metabolic and morphological changes similar to those occurring in human MI [7–10]. ISP, pharmacologically, is a *β*-adrenergic agonist and, chemically, is a synthetic catecholamine which upon subcutaneous injection at submaximal dose induces infarct like cell death of cardiac muscle in rodents [7]. Among several mechanisms proposed for ISP-induced MI, production of highly cytotoxic-free radicals through autooxidation and disturbed physiological balance between production of free radicals and antioxidative defense is widely accepted [23]. MI is characterized by cardiac dysfunction, lipid peroxidation, altered activities of cardiac injury markers, and depletion of endogenous antioxidants [9, 10]. Furthermore, it has also been documented that the heart is highly susceptible to oxidative stress compared to other tissues due to lower activity of antioxidant defense in the heart tissues [24]. The endogenous antioxidant defense network constitutes enzymatic (SOD and catalase) and nonenzymatic (GSH) molecules to neutralize the ROS mediated tissue injury in oxidative stress [23]. SOD catalyzes the dismutation of superoxide anions to oxygen and H_2_O_2_, which is further detoxified by catalase to water. The decrease in activities of SOD and catalase following ISP administration demonstrates overwhelming increase of free radicals, superoxide, and hydrogen peroxide which causes cellular injury. TMQ pretreatment prevented decline of the myocardial SOD activities in ISP administered rats. TMQ is widely reported as a potent antioxidant and found to protect against oxidative damage directly by reducing H_2_O_2_ to water and indirectly by increasing the levels of GSH [15–18, 21]. In agreement with previous reports [15–18], the present findings are strongly suggestive of potent antioxidant activity of TMQ against ISP-induced oxidative stress in MI.

In heart, endogenous antioxidant substrate, GSH, regulates cell function and provides protection by scavenging ROS like superoxide, peroxy radicals, and singlet oxygen. Reduction in the level of GSH following ISP administration shows its depletion to overcome the oxidative stress [10]. The improvements in GSH levels with TMQ treatment demonstrate their antioxidant and free radical scavenging activity in consonance with previous studies [6, 9, 10].

Lipid peroxidation is an important pathogenic event in MI and accumulation of oxidants makes the cell membranes more susceptible to oxidative injury and formation of lipid peroxidation product, MDA, that reflects the damage of the myocardial cell contents [6]. Altered membrane structure and enzyme inactivation in myocardial infarction is the most important event which is induced due to overproduction of MDA in ISP-induced myocardial injury and the increased production of free radicals might be responsible for the damage of cell membrane. The decrease in MDA level following pretreatment with TMQ can be ascribed to the enhanced activities of antioxidant status in myocardium. The antioxidant activity could be explained by its interaction with the mitochondrial respiratory chain, significant for the conversion of administered TMQ to hydroquinone. TMQ (oxidized form) possesses low antioxidant activity while its reduced form (thymohydroquinone) exerts a high radical scavenging capacity [25]. Several studies have demonstrated that TMQ efficiently scavenge free radicals and provides defense against lipid peroxidation [15, 16]. The antioxidant and free radical scavenging activity of TMQ revealed in present study corroborates with the earlier reports and demonstrates the antioxidant activity in ISP-induced MI associated with oxidative damage [15, 16].

Additionally, reduced availability of NO due to impairment in its synthesis and/or enhanced degradation by superoxide anion has been implicated as a major cause of oxidative stress in numerous cardiovascular diseases [26–28]. The complex oxidative milieu in myocardial ischemia triggers several pathophysiological mechanisms that simultaneously stimulate or suppress NO production. Numerous studies demonstrated that decrease in myocardial NO levels are due to enhanced oxidative stress and decreased NOS expression [26–28]. However, pretreatment with TMQ significantly increased NO levels in the heart. This effect is supported by the reduction of oxidative stress and can be ascribed to the induction of NOS following a counterbalance of NOS activity under the oxidative burst in accordance with previous studies [26, 28].

Several studies including ours have demonstrated that ISP administration produce marked hemodynamic alterations manifested in the form of systolic or diastolic dysfunction and increased heart rate [6, 9, 10]. The hemodynamic changes further lead to increased left ventricular wall thickness and ST-segment elevation. Black cumin containing TMQ has been reported to cause a dose-dependent decrease in the arterial blood pressure and heart rate in hypertensive rats mediated by several mechanisms including serotonergic, muscarinic, and adrenergic mechanisms [21, 29]. The adrenergic receptors stimulation due to overproduction of catecholamines is known to be a major cause of stress-induced cardiac dysfunction [30]. It has been well established that excessive plasma concentrations of catecholamines produce cardiac dysfunction [30]. This is also important in the purview of reports of development of combination therapeutics like atenolol and propranolol with* quercetin* or* Semecarpus* reported cardioprotective in ISP-induced MI compared to individual treatments [31]. Thus, TMQ can be used in treatment of MI as an adjuvant and needs to be investigated. In addition to these receptor dependent mechanisms, the ability of TMQ as a potent free radical scavenger and antioxidant may affect cardiac function and explicate its potential as a cardioprotective agent. In agreement with the previous studies [15, 16], the improved myocardial antioxidant status and energetics following TMQ treatment are presumed to translate into the recovery of cardiac functions altered during ISP-induced MI.

Myocardial cells contain several enzymes and macromolecules which on metabolic damage release the extracellular fluid and serve as diagnostic markers of myocardial injury [10]. The release of these enzymes reflects an alteration in the plasma membrane integrity and permeability in response to *β*-adrenergic stimulation [6]. Measuring AST, ALT, CK, and LDH activities is necessary to ascertain extent of myocardial injury. In present study, ISP administration caused a rise in the level of diagnostic marker enzymes AST, ALT, LDH, and CK due to leakage from tissue to blood serum as a result of damaged or destroyed cardiomyocytes, as well as, the cells damaged because of insufficient supply of oxygen and oxidative damage of myocardium which render the cell membrane fragile, porous, or ruptured. The increased levels of these enzymes are indicative of severity of cell necrosis and ISP mediated peroxidative myocyte injury. The serum levels of CK and LDH are early and late diagnostic markers of MI. CK level rises within 2 to 8 hrs of onset of MI and LDH begins to rise in 12 to 24 hrs of MI with a peak in 2-3 days. Treatment with TMQ reduced the serum levels of AST, ALT, CK, and LDH concomitant to the histopathological preservation in ISP administered rats. The histopathological preservation and inhibition of lipid peroxidation could be reasonable to correlate with the reduced leakage of myocardial enzymes in serum. It can be inferred that TMQ might have preserved cell integrity and stabilized the myocardial membrane which restricts the leakage of these marker enzymes from the heart into blood.

The protective effects of TMQ on histopathological changes of myocardium were further supported by light microscopic observations. Subsequent to ISP administration, significant myonecrosis, edema, and infiltration of inflammatory cells were observed in light microscopic examination of the myocardium. However, TMQ pretreatment to ISP-challenged rats has shown resistance towards necrosis, edema, and inflammation and protected cardiomyocytes from the deleterious effects of ISP. Rats which received TMQ* per se* treatment exhibited a normal myocardial histology, which is suggestive of the fact that TMQ at this dose does not render any significant adverse effects on myocardium and is safe for myocardial cells. In addition, the ratio of heart/body weight is an index of cardiac hypertrophy and a significant altered ratio indicates myocardial injury in ISP administered rats. However, treatment with TMQ to ischemic rats has significantly reduced cardiac hypertrophy as evidenced by reduction of heart/body weight.

Increasing evidences support that inflammation plays an important role in cardiovascular disease, including MI or silent myocardial ischemia and acute coronary syndromes [32, 33]. Several studies have described ISP stimulation induced myocardial proinflammatory cytokines, TNF-*α* and IL-1*β* expressions [34, 35]. On the other hand, studies have shown that *β*-adrenergic blockade treatment could exert beneficial effects on myocardial injury which is accompanied by selective reductions in myocardial expression of proinflammatory cytokines, IL-1*β*, IL-6, and TNF-*α* [36]. Therefore, inhibition of proinflammatory cytokines has been considered an important approach to protect myocardial damage [36, 37]. Studies suggest that proinflammatory cytokines act as pleiotropic polypeptides that are independently associated with inflammation and oxidative stress and release of these cytokines leads to myocardial injury through several mechanisms [38]. Among these cytokines, TNF-*α* triggers the release of other proinflammatory cytokines and influences neutrophils recruitment which further results in cell death [38]. In the present study, a significant increase in levels of IL-1*β*, IL-6, and TNF-*α*, in heart tissues of rats injected ISP, is in agreement with previous studies [34, 35]. Following pretreatment with TMQ, significant reduction in the level of proinflammatory cytokines is clearly suggestive of its anti-inflammatory effect in ischemic heart. In several studies, TQ has been shown to be effective in improving oxidant-antioxidant balance and reduced the levels of proinflammatory mediators (IL-1*β*, IL-6, TNF-*α*, IFN-*γ*, and PGE_2_) and modulates several molecular pathways mediated by TNF-*α* [17, 39, 40].

Several anti-inflammatory mechanisms of TMQ have been reported including its role in regulation of peroxisome proliferator-activated receptor-gamma (PPAR-*γ*) activation [41], which plays an important role in the regulation of a variety of biological processes within the cardiovascular system [42]. Various PPAR-*γ* agonists have been shown to reduce myocardial injury in animal models by inhibiting proinflammatory cytokines, modulating redox signaling, and upregulating prosurvival signaling [3, 6, 39]. The advantage of natural ligands of PPAR-*γ* over synthetic ones has garnered attention in recent years due to multiple pharmacological benefits with lesser adverse effects. Integrating altogether the multiple bioactivities including anti-inflammatory, antioxidant, immunomodulatory, it is plausible to speculate that this multimodal anti-inflammatory and antioxidant mechanism may underlie the cardioprotective effect of naturally occurring molecule, TMQ.

## 5. Conclusion

Based on our present findings, it can be concluded that TMQ exhibits its cardioprotective effects by enhancing antioxidant defense, inhibiting lipid peroxidation, and inflammation as well as preserving the cardiomyocytes, which all together may translate into the functional recovery of heart function.

## Figures and Tables

**Table 1:** Histopathological changes in rat myocardium of different experimental groups.

Treatment groups	Myonecrosis	Inflammation	Edema
Control	—	—	—
ISP	+++	+++	+++
TMQ 20 mg/kg	—	—	—
TMQ 20 mg/kg + ISP	+	+	+

—: absence of inflammation, edema, and necrosis; +: focal areas of inflammation, edema, and necrosis; +++: confluent areas of inflammation, edema, and necrosis.
